# Developing a genetic engineering method for *Acetobacterium wieringae* to expand one-carbon valorization pathways

**DOI:** 10.1186/s13068-023-02259-6

**Published:** 2023-02-14

**Authors:** João P. C. Moreira, John T. Heap, Joana I. Alves, Lucília Domingues

**Affiliations:** 1 CEB - Centre of Biological Engineering, University of Minho, 4710-057 Braga, Portugal; 2 LABBELS - Associate Laboratory, Braga/Guimarães, Portugal; 3 School of Life Sciences, University of Nottingham, Biodiscovery Institute, University Park, Nottingham, NG7 2RD UK

**Keywords:** Syngas, Carbon monoxide, Biofuels, Chemicals, Acetogen, *Acetobacterium*, Transformation, Electroporation, Genetic engineering

## Abstract

**Background:**

Developing new bioprocesses to produce chemicals and fuels with reduced production costs will greatly facilitate the replacement of fossil-based raw materials. In most fermentation bioprocesses, the feedstock usually represents the highest cost, which becomes the target for cost reduction. Additionally, the biorefinery concept advocates revenue growth from the production of several compounds using the same feedstock. Taken together, the production of bio commodities from low-cost gas streams containing CO, CO_2_, and H_2_, obtained from the gasification of any carbon-containing waste streams or off-gases from heavy industry (steel mills, processing plants, or refineries), embodies an opportunity for affordable and renewable chemical production. To achieve this, by studying non-model autotrophic acetogens, current limitations concerning low growth rates, toxicity by gas streams, and low productivity may be overcome. The *Acetobacterium wieringae* strain JM is a novel autotrophic acetogen that is capable of producing acetate and ethanol. It exhibits faster growth rates on various gaseous compounds, including carbon monoxide, compared to other *Acetobacterium* species, making it potentially useful for industrial applications. The species *A. wieringae* has not been genetically modified, therefore developing a genetic engineering method is important for expanding its product portfolio from gas fermentation and overall improving the characteristics of this acetogen for industrial demands.

**Results:**

This work reports the development and optimization of an electrotransformation protocol for *A. wieringae* strain JM, which can also be used in *A. wieringae* DSM 1911, and *A. woodii* DSM 1030. We also show the functionality of the thiamphenicol resistance marker, *catP*, and the functionality of the origins of replication pBP1, pCB102, pCD6, and pIM13 in all tested *Acetobacterium* strains, with transformation efficiencies of up to 2.0 × 10^3^ CFU/μg_DNA_. Key factors affecting electrotransformation efficiency include OD_600_ of cell harvesting, pH of resuspension buffer, the field strength of the electric pulse, and plasmid amount. Using this method, the acetone production operon from *Clostridium acetobutylicum* was efficiently introduced in all tested *Acetobacterium* spp., leading to non-native biochemical acetone production via plasmid-based expression.

**Conclusions:**

*A. wieringae* can be electrotransformed at high efficiency using different plasmids with different replication origins. The electrotransformation procedure and tools reported here unlock the genetic and metabolic manipulation of the biotechnologically relevant *A.* *wieringae* strains. For the first time, non-native acetone production is shown in *A. wieringae*.

**Supplementary Information:**

The online version contains supplementary material available at 10.1186/s13068-023-02259-6.

## Background

The development of new processes for sustainable energy and chemical production is in great demand due to the urge of reducing the carbon footprint, aiming to fix current environmental issues [1]. With raised climate ambitions to reduce at least 55% (from 1990 levels) of EU greenhouse gas (GHG) emissions by 2030 [2] and targets on climate and energy framework of at least 32% share for renewable energy [3], it is crucial to develop new technologies for generating renewable energy. While electricity seems to be the solution for some cases, such as road transportation, it is unlikely that electrical power alone will be able to replace fossil fuels as a platform for global chemical needs. Instead, for the chemical industry, heavy road transport, marine, and aviation sectors, bioprocesses are one of the few options to replace the current fossil feedstock with a renewable resource, thereby reducing the sectors’ GHG emissions [4, 5].

One promising emerging technology to address these challenges is to source low-carbon fuels and chemicals via fermentation of syngas (CO, CO_2_, H_2)_, which can be obtained after gasification of renewable resources such as lignocellulosic biomass and also from industrial and municipal wastes [6], and as a by-product of industries like steel manufacturing [7]. Syngas fermentation is based on the ability of microorganisms to convert syngas components into alcohols and carboxylic acids [8, 9]. Most of these microorganisms are autotrophic acetogens that can fix CO/CO_2_ and H_2_ via the Wood–Ljungdahl pathway [10, 11]. Recent progress in genetic and metabolic engineering of acetogens [12–14] offers gateways to produce a wide variety of non-natural compounds through the introduction of exogenous pathways that are either borrowed from other organisms or synthetically designed. The autotrophic acetogens with developed genetic toolkits are mostly *Clostridium ljungdahlii*, *Clostridium autoethanogenum*, *Clostridium carboxidivorans*, *Eubacterium limosum*, *Moorella thermoacetica, Thermoanaerobacterium kivui,* and *Acetobacterium woodii* [12, 14]. Examples of production of non-native compounds in these organisms are butanol, butyrate, acetone, mevalonate, and isoprene production in *C. ljungdahlii* [15–18], or acetone, isopropanol, isobutanol, and butyrate in *A. woodii* [19–22]. *C. autoethanogenum* was also manipulated for the production of acetone and isopropanol from syngas, which could be achieved at industrial pilot scale [23]. Most used genetic parts for these organisms such as resistance genes and origins of replication are functional in multiple species [12] and thereby expanding the existing molecular tools to other acetogens should be possible. The currently studied bioprocesses involving gas fermentation usually face limitations concerning low growth rates, toxicity by gas streams, and low productivity [24]. By studying non-model organisms with different characteristics, these limitations may be overcome. In previous work, a novel acetogenic bacterium strain, *Acetobacterium wieringae* strain JM, was isolated by our research team [25]. This novel autotrophic acetogen is an interesting organism with different physiology when compared to its closest relative *A. woodii*. It is very versatile, having industrially relevant characteristics since it grows at high rates in different gas compositions and does not require additional carbon sources as supplementation [25]. Additionally, as shown in genomic analysis, non-model organisms within the *Acetobacterium* genus may provide varied metabolic capabilities and other abilities to survive in diverse environments and potentially be exploited for biotechnological applications [26]. *A. wieringae* JM produces mainly acetate and ethanol from gas fermentation [25]. With an interest in expanding the product frame of gas fermentation, *A. wieringae* JM was previously used in a co-cultivation strategy to produce propionate from carbon monoxide [27]. To further expand the product frame of *A. wieringae* JM, we aim to make this acetogen genetically accessible and implement heterologous pathways to produce more valuable compounds.

Here, we report the development of a robust, stable, and efficient electroporation-mediated transformation system for *A. wieringae* JM which can also be used in other *Acetobacterium* strains. We determine the impact of several parameters of the transformation procedure on the transformation efficiency, including different origins of replication. Furthermore, we demonstrate the expression of heterologous genes for acetone production.

## Results

### Screening of molecular tools for transformation of *A. wieringae* strain JM

To transform the novel acetogen *A. wieringae* strain JM, as commonly carried out for new potential hosts, we performed minimum inhibitory concentrations (MIC) assays to test the natural antibiotic resistance of *A. wieringae* JM to thiamphenicol, clarithromycin, spectinomycin, and tetracycline (Table 1). Strain JM did not show resistance to any of the tested antibiotics, so we decided to select the *catP* resistance marker to thiamphenicol at a working concentration of 15 μg/mL for the selection of *A. wieringae* JM transformants as the MIC was 3 μg/mL.

**Table 1:** Minimum inhibitory concentrations of four antibiotics in *A. wieringae* strain JM and typical working concentrations for the resistance markers of these antibiotics in *A. woodii* and *C. ljungdahlii*

Antibiotic	MIC (μg/mL) *A. wieringae* strain JM	Resistance marker gene	Working concentrations (μg/mL)	
			*A. woodii* DSM 1030	*C. ljungdahlii* DSM 13528
Thiamphenicol	3	*catP*	7.5 to 25	5 to 20
Clarithromycin	0.75	*ermB*/*ermC*	2.5 to 5	4 to 6
Spectinomycin	75	*aad9*	–	–
Tetracycline	0.75	*tetA*	10 to 20	–

A series of plasmids from the pMTL80000 system of modular *E. coli*–*Clostridium* shuttle vectors [28] with proven efficiency in *A. woodii* [19] were used to transform *A. wieringae* JM. They differ in their Gram^+^ origins of replication: pMTL82151 (pBP1 from *C. botulinum*); pMTL83151 (pCB102 from *C. butyricum*); pMTL84151 (pCD6 from *C. difficile*); and pMTL85141 (pIM13 from *Bacillus subtilis*) [28]. Firstly, we attempted the conjugative plasmid transfer using the conjugal donor strain *Escherichia coli* CA434. Such a conjugation system works well for many organisms and is particularly useful when no electroporation protocol is available. In contrast with some *Clostridium* species where this method was applied [28], *A. wieringae* JM is not resistant to D-cycloserine at the working concentration of 250 μg/ml, which is typically used as counterselection against the *E. coli* donor strain. Therefore, we tried an alternative approach to select transconjugants, using 25 μg/ml of thiamphenicol to select for the plasmid and autotrophic conditions (carbon and energy source: 60% CO, 30% H_2_, 10% CO_2_; (v/v)) to select for *A. wieringae* JM, also omitting yeast extract from the medium. The resulting cultures of the conjugative mating process of *E. coli* CA434 (pMTL83151 and pMTL84151) with *A. wieringae* JM could be grown after four consecutive transfers on selective mineral media under autotrophic conditions, but transconjugants of *A. wieringae* JM could not be identified nor isolated as *E. coli* could grow in symbiosis with *A. wieringae* JM under autotrophic conditions.

### Development of an electrotransformation procedure for *A. wieringae* strain JM

As the conjugation procedure failed to produce isolated transformants of *A. wieringae* JM, we developed a new protocol that can be used to transform *A. wieringae* JM, A. *wieringae*^T^, and A. *woodii*^T^, based on previously reported electrotransformation procedures for *A. woodii*. Table 2 lists the detailed differences between the published transformation protocols for *A. woodii* and the final version of our new procedure (protocol 7, Table 2). The first electrotransformation of *A. woodii* was published in 1994 by Strätz et al*.* [29] (protocol 1, Table 2). The transformed plasmids pMS3 and pMS4p carried the replicon derived from the plasmid pAMβ1. In that study, the resulting transformation efficiencies (E_T_), using tetracycline selection, were 4.5 × 10^3^ CFU/μg_DNA_ for both plasmids [29]. Publications using other procedures reported the successful usage of the following vectors although transformation efficiencies were not given: pJIR750 plasmids carrying the replicon pIP404 from *C. perfringens* [30] (protocols 3 and 4, Table 2); pMTL82151 (pBP1) (protocol 4, Table 2); pMTL83151 (pCB102) (protocols 2, 4 and 5, Table 2); and pMTL84151 (pCD6) (protocols 2 and 4, Table 2). In *C. ljungdahlii*, the utilization of protocol 2 (Table 2) with the plasmids pMTL82151 (pBP1) and pMTL83151 (pCB102) resulted in E_T_ values of 3.8 × 10^3^ and 3.1 × 10^3^ CFU/μg_DNA_, respectively [31]. More recently, Baker et al*.* reported a new transformation protocol (protocol 6, Table 2) for *A. woodii* using the plasmid pMTL83141 (pCB102) with E_T_ values of 4.0 × 10^5^ CFU/μg [32].

**Table 2:** Comparison of published electrotransformation procedures used in *Acetobacterium woodii* and a new procedure for *Acetobacterium* strains

	Strätz, 1994	Leang, 2013	Straub, 2014	Hoffmeister, 2016	Weitz, 2021	Baker, 2022	New protocol
Protocol	1	2	3	4	5	6	7
References	[29]	[22, 31, 33–38]	[39]	[19, 20, 40, 41]	[21]	[32]	This work
Transformed organisms	*A. woodii*^T^	*A. woodii*^T^ *C. ljungdahlii*^T^	*A. woodii*^T^	*A. woodii*^T^	*A. woodii*^T^	*A. woodii*^*T*^ *A. woodii* lab-adapted strain	*A. woodii*^T^ *A. wieringae*^T^ *A. wieringae* JM
Preparation of cells and plasmid							
Cell weakening agent on growth media	NI^a^	40 mM DL-threonine	20 mM DL-threonine	40 mM DL-threonine	40 mM DL-threonine	NI^a^	40 mM D-threonine
OD_600_ (Harvesting)	0.5	0.2–0.3	0.4–0.6	0.3–0.5	0.3–0.7	0.2–0.4	0.3–0.6
Centrifugation steps	Outside of chamber	Outside of chamber	Inside of chamber	Outside of chamber	Inside of chamber	Inside of chamber	Inside of chamber
Anaerobic chamber atmosphere	NI	83% N_2_, 7% H_2_, 10% CO_2_	NI	95% N_2_, 5% H_2_	95% N_2_, 5% H_2_	80% N_2_, 10% H_2_, 10% CO_2_	95% N_2_, 5% H_2_
Wash-buffer	270 mM sucrose	SMP^b1^	SMP^b1^	SMP^b1^	SMP^b1^	SMP^b2^	SMP^b1^
pH wash-buffer	NI	6.0	6.0	6.0	6.0	5.8	6.0
Washing steps	2	2	2	2	2	2	1–2
Resuspension buffer	270 mM sucrose	SMP^b1^ 10% DMSO^c^	SMP^b1^	SMP^b1^ 10% DMSO	SMP^b1^ 10% DMF^d^	SMP^b2^ 15% DMSO	SMP^b1^ 10% DMF
pH of res. buffer	NI	6.0	6.0	6.0	6.0	5.8	6.0
Cell density after resuspension [OD_600_]	25	200–300	30–50	300–500	40–100	30 – 60	30
Freeze − 80 °C	No	Yes	No	Yes	Yes	Yes	Yes
Plasmid methylation	Dcm^+e^ Dam^+f^	Dcm^−^ Dam^+^	Dcm^+^ Dam^+^, pACYC184–methyltransferase gene of *C. ljungdahlii*	Dcm^+^ Dam^+^	Dcm^+^ Dam^+^	Dcm^+^ Dam^+^	Dcm^+^ Dam^+^
*E. coli* strain for plasmid-prep	XL1-Blue (Stratagene)	B strain (NEB express)	XL1-Blue MRF' (Stratagene) pMCljS (CLJU_c03310)	XL1-Blue MRF' (Stratagene)	XL1-Blue MRF' (Stratagene)	HB101 (Promega)	TOP10 (Invitrogen)
Electroporation process							
Preincubation with plasmid on ice	5 min	No	No	No	1 min	Yes	1–2 min
Volume of cells [µL]	40	25	600	25	25	100	200
Plasmid amount [µg]	0.40	1–5	1	1–5	3–5	1	1–3
Electroporation cuvettes gap [cm]	0.2	0.1	0.4	0.1	0.1	0.2	0.2
Cell-plasmid ratio (OD_600_ × mL/µg)	2.5	1–7.5	18–30	1.5–12.5	0.2–0.8	3–6	2–6
Electric pulse	10 kV 400Ω 25μF	0.625 kV 600Ω 25μF	2.5 kV 600Ω 25μF	0.625 kV 600Ω 25μF	0.625 kV 600Ω 25μF	1.0 kV 200Ω 50μF	2.0 kV 20Ω 25μF
Electric field strength [kV/cm]	50	6.25	6.25	6.25	6.25	5	10
Electroporator	NI	Gene Pulser Xcell (Bio-Rad)	NI	Gene Pulser Xcell (Bio-Rad)	Gene Pulser Xcell (Bio-Rad)	Gene Pulser Xcell (Bio-Rad)	*E. coli* Pulser (Bio-Rad)
Incubation after electroporation on ice	5 min	–	–	–	–	–	–
Cultivation after electroporation	0.960 mL GPM broth	10 mL PETC 1754 10 g/L fructose	5 mL modified DSMZ 135 10 g/L fructose	5 mL modified DSMZ 135 3 g/L fructose	5 mL modified PETC 1754 7 g/L fructose	10 mL modified DSMZ 135 3.6 g/L fructose	3 mL basal media 10 g/L fructose
Recovery cultivation time	7 h	9–12 h	3 days	> 12 h	Till doubling then antibiotic was added until growth was observed	5 h	20 h
Plating	Liquid culture on solid agar (Petri dish)	Liquid culture mixed with molten agar (Petri dish)	Liquid culture	Liquid culture	Liquid culture on solid agar (Petri dish)	Liquid culture on solid agar (Petri dish)	Liquid culture mixed with molten agar (serum bottle)
Antibiotic [µg/mL]	Tetracycline 10	Thiamphenicol 5	Thiamphenicol 10	Thiamphenicol 10	Thiamphenicol 10	Thiamphenicol 12.5	Thiamphenicol 15
Incubation	NI	Inside of chamber	NI	Inside of chamber	Inside of chamber	Inside of chamber	Outside of chamber
Plasmids (Gram^+^ replicon)	pMS3, pMS4 (pAMβ1)	pMTL_8312 (pCB102), pMTL84151 (pCD6)	pJIR750 (pIP404)	pJIR750 (pIP404), pMTL82151 (pBP1), pMTL83151 (pCB102), pMTL84151 (pCD6)	pMTL83151 (pCB102)	pMTL83141 (pCB102)	pMTL82151 (pBP1), pMTL83151 (pCB102), pMTL84151 (pCD6), pMTL85151 (pIM13)

^a^NI not indicated, ^b1^SMP 270 mM sucrose, 1 mM MgCl_2_, 7 mM NaH_2_PO_4_, ^b2^SMP 270 mM sucrose, 1 mM MgCl_2_, 1 mM NaH_2_PO_4_, ^c^DMSO dimethyl sulfoxide, ^d^DMF dimethylformamide, ^e^Dcm cytosine methyltransferase, ^f^Dam adenine methyltransferase, ^T^ type strain

The most important changes for higher efficiency and reproducibility were the use of fixed cell density of competent cells resulting in electroporation cell-plasmid ratios of 2 to 3 (OD_600_ × mL/µg), the use of electric field strength of 10 kV/cm, and the selection of transformants in serum anaerobic bottles with molten agar (Additional file 1 Fig. S1a). Furthermore, during competent cell preparation, cells are usually grown with cell wall-weakening agents, such as lysozyme, glycine, DL-threonine, or penicillin G [42, 43], to promote the passage of DNA through the thick Gram^+^ cell wall. Protocols 2 to 5 report the use of DL-threonine (20–40 mM) during cell growth before the preparation of competent cells (Table 2). In our hands, *A. wieringae* JM could not be transformed without the use of cell wall-weakening agents. The use of 40 mM D-threonine allowed the transformation of *A. wieringae* JM while the addition of 1.25% glycine (% *w*/*v*) caused growth inhibition. Protocols 1 and 3 do not allow storage of competent cells before electroporation, while protocols 2, 4, and 5 use DMSO, protocol 5 uses DMF in the SMP resuspension buffer as cryoprotectants to allow storage of competent cells. We used DMF as it is also utilized as a solvent during the preparation of the antibiotic thiamphenicol. In our protocol, the plasmid DNA was prepared from *E. coli* TOP10 (Invitrogen, MA, USA) cells that have the methyltransferases Dam and Dcm which methylate the DNA bases adenine and cytosine, respectively, allowing protection against endonucleases. No external, site-specific methylation was used, in contrast with protocol 3 where a methylase gene of *C. ljungdahlii* (CLJU_c03310) was used in addition to an *E. coli* Dam^+^/Dcm^+^ background. Without this, the transformation of *A. woodii* with pJIR750 would fail [39]. Protocols 1, 4, and 5 report the utilization of Dam^+^/Dcm^+^ methylation background with *E. coli* XL1-Blue (MRF’) strain (Stratagene, CA, USA), while protocol 2 utilizes an *E. coli* strain lacking Dcm (New England Biolabs, MA, USA) which yielded better transformation results in *C. ljungdahlii* [31].

**Fig. 1:**
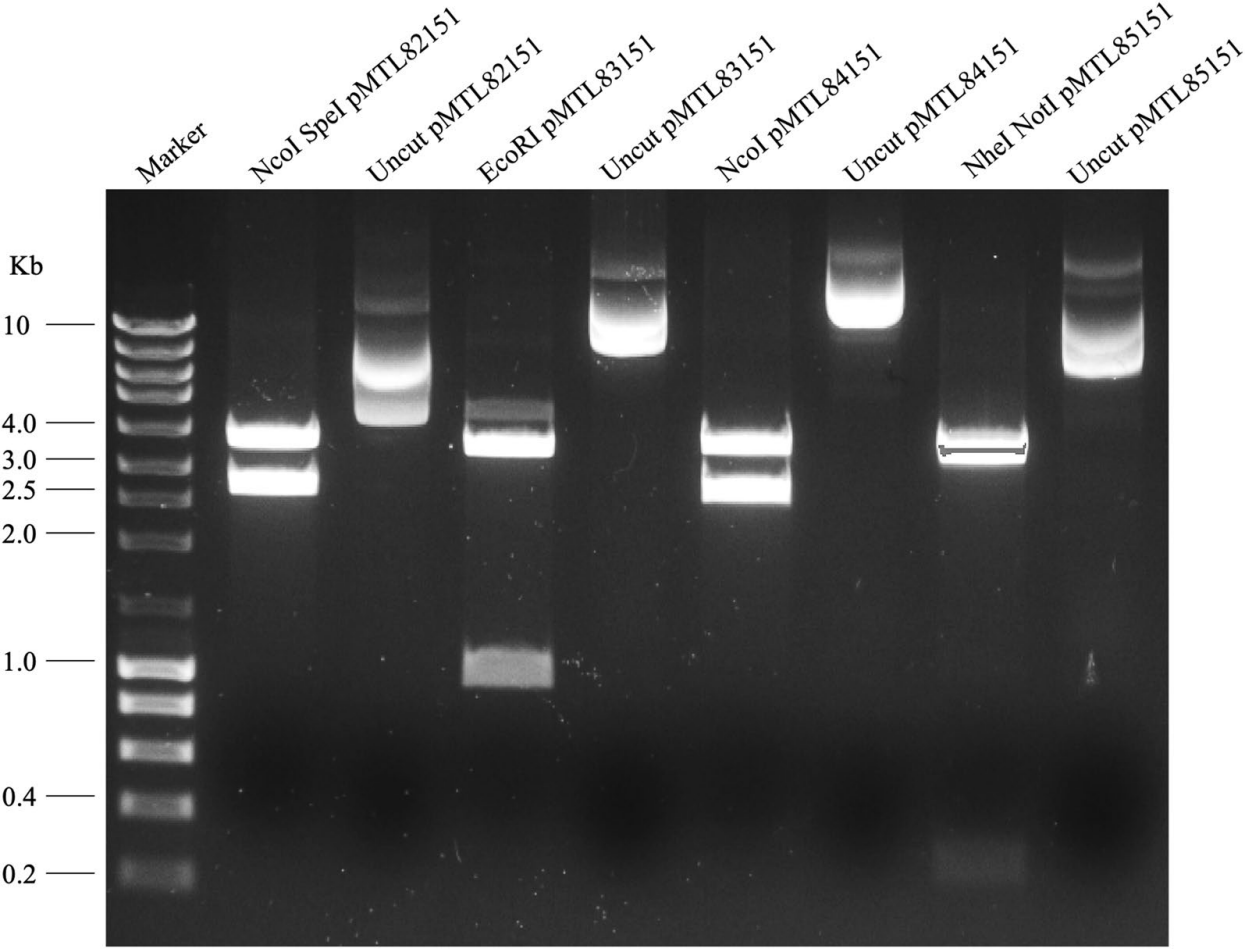
Plasmid restriction analysis of *Acetobacterium wieringae* strain JM transformants. Digestion of pMTL82151, pMTL83151, pMTL84151, and pMTL85151, using NcoI-HF and SpeI-HF, EcoRI-HF, NcoI-HF, NheI-HF, and NotI-HF, respectively, resolved on a 1% agarose gel. Digestion reactions contained 0.5 μg of plasmid DNA and 1 μL of restriction enzymes in a total volume of 50 μL. For comparison, plasmids are shown undigested and digested. Expected band sizes are: 3114 and 2140 bp, pMTL82151; 3475 and 1001 bp, pMTL83151; 3606 and 2697 bp, pMTL84151; 3453 and 276 bp, pMTL85151

To confirm the presence of the pMTL80000 plasmids in *A. wieringae* JM transformants, we isolated plasmid DNA from recombinant cells and transformed it into *E. coli* DH5α cells. After re-purification, the pMTL80000 vectors were verified by restriction analysis. Correct digestion profiles were obtained for each re-purified plasmid (Fig. 1), confirming the presence of the transformed plasmids in *A. wieringae* JM.

**Fig. 2:**
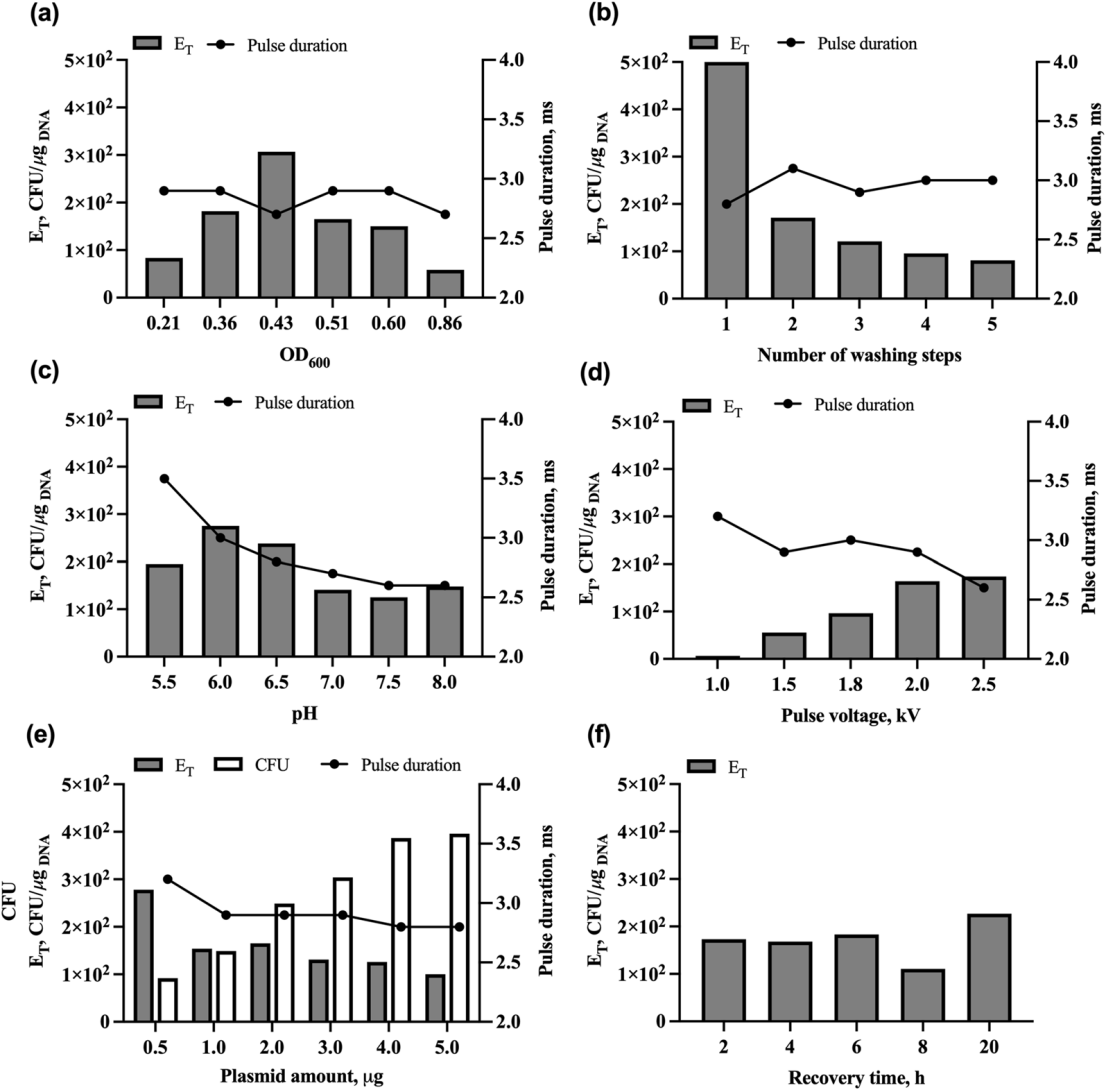
Optimization of electrotransformation procedure in *Acetobacterium wieringae* strain JM using the plasmid pMTL83151. **a** Effect of harvesting OD_600_ during the preparation of *A. wieringae* JM competent cells on transformation efficiency, E_T_. Growing cells were sampled during exponential phase (OD_600_ 0.21, 0.36, 0.43, 0.51, 0.60, 0.86) for preparation of competent cells prior electroporation. After resuspension of competent cells in SMP 10% DMF, all conditions had an OD_600_ of 30. **b** Effect of the number of washing steps during preparation of *A. wieringae* JM competent cells on transformation efficiency. Cells were grown to OD_600_ of 0.36 and were sampled to be washed 1, 2, 3, 4, and 5 times with SMP buffer during the preparation of competent cells before electroporation. **c** Effect of pH of resuspension buffer on transformation efficiency. Cells were grown to OD_600_ of 0.40, washed in SMP buffer pH 6, and were sampled to be resuspended in SMP 10% DMF pH 5.5, 6.0, 6.5, 7.0, 7.5, and 8.0 during competent cell preparation before electroporation. **d** Effect of pulse voltage (field strength) on transformation efficiency. E_T_ was measured using electric pulses of 1.0, 1.5, 1.8, 2.0, and 2.5 kV, corresponding to field strengths of 5.0, 7.5, 8.0, 10, and 12.5 kV/cm. **e** Effect of plasmid DNA amount on colony-forming units (CFU) and transformation efficiency. Separately, 0.5, 1.0, 2.0, 3.0, 4.0, and 5.0 μg of plasmid corresponding, respectively, to cell–plasmid ratios of 12.0, 6.0, 3.0, 2.0, 1.5, and 1.2, were added to competent cells of *A. wieringae* JM and electroporated. The total number of colony-forming units and transformation efficiency was quantified. **f** Effect of post-electroporation incubation time on transformation efficiency. Cells were electroporated, transferred to a 3 mL outgrowth medium, and incubated for 2, 4, 6, 8, and 20 h before selective plating

The development and optimization of our electrotransformation procedure for maximized efficiency in *A. wieringae* JM required the empirical study of parameters involved in the preparation of electrocompetent cells such as the density at which cells are harvested, number of washing steps, pH of resuspension buffer, and the study of key aspects of the electroporation process such as pulse voltage, plasmid amount, and recovery time. The plasmid pMTL83151 carrying the pCB102 replicon yielded the most colonies during the first tests, therefore it was used for the optimization experiments, results are shown in Fig. 2.

When preparing competent cells, the cell density at which cells are harvested can impact the transformation efficiency [44]. The published transformation protocols for *A. woodii* harvested cells when OD_600_ reached 0.2–0.7 (protocols 1–6, Table 2). We harvested *A. wieringae* JM at six different optical densities (OD_600_ of 0.21, 0.36, 0.43, 0.51, 0.60, and 0.86). The cells harvested at OD_600_ of 0.43 produced the most colonies, resulting in transformation efficiency (E_T_) of 3.1 × 10^2^ CFU/μg_DNA_. Cells harvested at OD_600_ of 0.36, 0.51, and 0.60 resulted in the relative reduction of E_T_ by 0.41- to 0.51-fold, while harvesting cells early or late during the exponential phase of growth (OD_600_ of 0.21 and 0.86), reduced E_T_ by 0.73- and 0.81-fold, respectively (Fig. 2a). Therefore, we defined the optimal harvesting OD_600_ range of 0.3–0.6 (protocol 7, Table 2).

All available transformation protocols report the washing of competent cells in two steps (protocols 1 – 5, Table 2). We attempted to increase the E_T_ of *A. wieringae* JM by increasing the number of washing steps up to 5. However, maximal E_T_ was obtained with only one washing step (5.0 × 10^2^ CFU/μg_DNA_, Fig. 2b). Washing the cells two times resulted in a reduction of E_T_ by 0.66-fold, while five washes had an even higher impact on E_T_, by 0.84-fold. As centrifugation of competent cells is performed inside the anaerobic chamber, the refrigeration efficiency decreases as the time of centrifugation increases. Therefore, increasing the number of washes compromises the E_T_ of *A. wieringae* JM.

Cultivation media *for A. wieringae* JM and *A. woodii* usually have pH close to neutral (~ 6.8–7.0), but transformation procedures for *A. woodii* report the use of pH 5.8–6.0 on the washing and resuspension buffers (protocols 1–6, Table 2). We tested the effect of pH (range of 5.5–8.0) in the SMP 10% DMF resuspension buffer during the preparation of competent cells on E_T_ of *A. wieringae* JM (Fig. 2c). Results show that the use of pH 6.0 and 6.5 produced the most colonies, resulting in E_T_ values of 2.8 × 10^2^ and 2.4 × 10^2^ CFU/μg_DNA_, respectively. The more acidic pH of 5.5 reduced E_T_ by 0.29-fold, while pH of 7.0, 7.5, and 8.0 caused reductions in E_T_ of more than 0.56-fold. The pulse duration decreased with the increment of pH, without a significant impact on E_T_.

Key electroporation parameters, such as voltage, resistance, or field strength, which are dependent on cuvette gap width, influence the transformation efficiency. Other reported procedures for *A. woodii* use different combinations of cuvette gap width and pulse voltage, resulting in a pulse strength of 6.25 kV/cm with 600 Ω of resistance in protocols 2–5, and 50 kV/cm in protocol 1 with 400 Ω of resistance (Table 2). We investigated the effects of the electrical pulse concerning voltage (i.e., field strength), using cuvettes with 0.2 cm gap width, as the electroporation device used (*E. coli* Pulser^™^ Transformation Apparatus) only allows adjustment on pulse voltage. The electric resistance of this electroporation device is 20 Ω. Pulses of 1.0, 1.5, 1.8, 2.0, and 2.5 kV were administered, corresponding, respectively, to field strengths of 5.0, 7.5, 8.0, 10, and 12.5 kV/cm (Fig. 2d). Higher voltage resulted in higher transformation efficiency, the voltage of 2.5 kV was found to produce the greatest E_T_ values (1.7 × 10^2^ CFU/μg_DNA_) but sample ‘arcing’ occurred often, although pulse of 2.0 kV only slightly reduced E_T_ by 0.06-fold without any ‘arcing’ occurrence. With the weakest tested pulse, 1.0 kV, it was still possible to obtain colonies but there was a 0.98-fold reduction in E_T_. 1.5 and 1.8 kV impacted E_T_ by 0.68 and 0.45-fold, respectively. Pulse duration decreased by increasing pulse voltage.

Plasmid amount and cell–plasmid ratio (OD_600_ × mL/µg) can also influence the transformation efficiency. In reported transformation procedures for *A. woodii*, there is some variation in used plasmid amounts and the cell–plasmid ratios, between and within different protocols (Table 2). Plasmid amounts of 0.5, 1.0, 2.0, 3.0, 4.0, and 5.0 µg were used, corresponding, respectively, to cell-plasmid ratios of 12.0, 6.0, 3.0, 2.0, 1.5, and 1.2 (Fig. 2e). Although the total number of transformants was found to increase as expected between 0.5 and 5.0 μg of pMTL83141, the greatest E_T_ occurred at the lowest quantity of DNA tested, 0.5 μg of plasmid DNA (2.8 × 10^2^ CFU/μg_DNA_), but less than 100 colonies were observed in duplicates. By using 1–3 μg of plasmid, ~ 150–300 colonies were found which resulted in the average E_T_ of 2.3 × 10^2^ (± 7.9 × 10^1^) CFU/μg_DNA_.

The recovery period after applying an electric field to the cells is performed in a liquid medium with no selective pressure and can also influence the transformation efficiency as longer periods may lead to plasmid loss and shorter periods may not allow the expression of the selection marker gene before plating. Recovery periods of 5 h up to 3 days have been reported for *A. woodii* which may also depend on the used recovery media and Gram^+^ replicon, which also differs between protocols (Table 2). For assessing outgrowth duration with pMTL83151 in *A. wieringae* JM, electroporated cells were incubated for 2, 4, 6, 8, and 20 h before plating on a selective medium. Transformants could be obtained in all conditions, 20 h of recovery time resulted in the greatest E_T_ value of 2.3 × 10^2^ CFU/μg_DNA_, but there was not a significant difference for the recovery periods of 2, 4, and 6 h, representing on average a 0.23 (± 0.03)-fold reduction, 8 h of recovery time had the highest impact on E_T_, with a 0.51-fold reduction (Fig. 2f).

### Application of the electrotransformation protocol to other vectors and strains

**Fig. 3:**
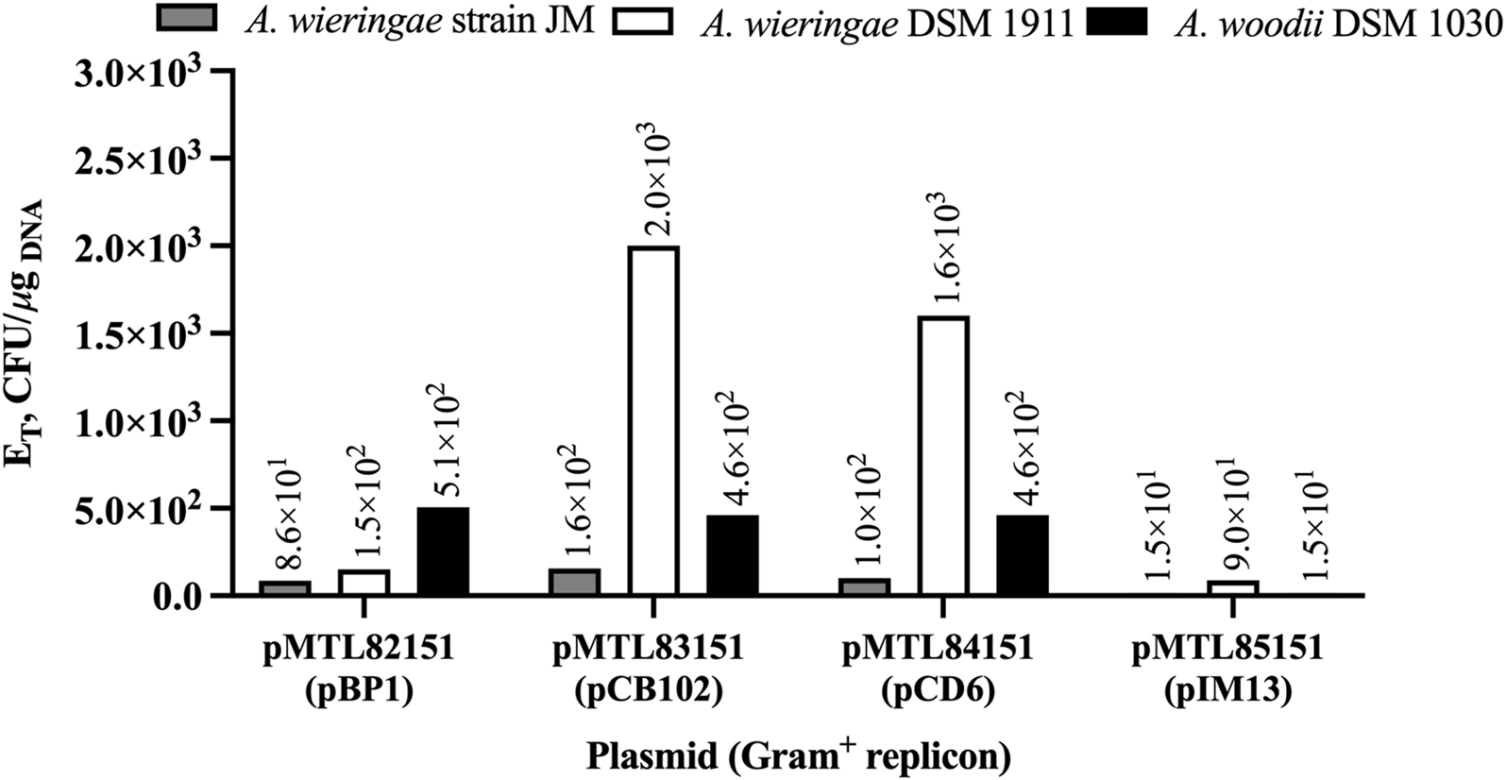
Transformation efficiencies for different *Acetobacterium* strains and different origins of replication. *A. wieringae* strain JM, *A. wieringae* DSM 1911^ T^, and *A. woodii* DSM 1030^ T^ were transformed with the plasmids pMTL82151 (pBP1), pMTL83151 (pCB102), pMTL84151 (pCD6), and pMTL85151 (pIM13)

The effect of the replicons pBP1, pCB102, pCD6, and pIM13 on the transformation efficiency of *A. wieringae* JM, *A. wieringae*^T^, and *A. woodii*^T^, was assessed using our optimized transformation protocol (protocol 7, Table 2). All *Acetobacterium* strains were harvested at OD_600_ of ~ 0.30, washed two times during the preparation of competent cells, and 3 μg of plasmid were used for electroporation. All vectors carrying the different replicons have the same backbone including the *catP* resistance gene but differ in size: 5254 bp, pMTL82151 (pBP1); 4476 bp, pMTL83151 (pCB102); 6297 bp, pMTL84151 (pCD6); 3729 bp, pMTL85151 (pIM13). Colonies could be obtained using the four different vectors in all tested *Acetobacterium* strains, and results show that E_T_ differences between plasmids are not justified by plasmid size, but by the replication mechanism or stability of each replicon (Fig. 3). In *A. wieringae* JM, pCB102 produced the most colonies, with an E_T_ of 1.6 × 10^2^ CFU/μg_DNA_, while E_T_ of pCD6, pBP1, and pIM13 were, respectively, 0.38-, 0.46-, and 0.90-fold lower (Fig. 3). In *A. wieringae*^T^, the use of pCB102 also had the highest E_T_ of 2.0 × 10^3^ CFU/μg_DNA_, while E_T_ of pCD6, pBP1, and pIM13 were, respectively, 0.20-, 0.93-, and 0.96-fold lower (Fig. 3). The transformation efficiency of *A. wieringae*^T^ with pCB102 was 12.3-fold higher than the E_T_ of *A. wieringae* JM for the same replicon. Other replicons also resulted in higher E_T_ with *A. wieringae*^T^ than with *A. wieringae* JM. Lastly, in *A. woodii*^T^, transformation efficiencies were very similar for the replicons pBP1, pCB102, and pCD6, with an average E_T_ of 4.8 × 10^2^ (± 2.9 × 10^1^) CFU/μg_DNA_, while E_T_ of pIM13 was 0.97-fold lower (Fig. 3). For all tested plasmids, transformation efficiencies in *A. woodii* were equal (pIM13) or higher (pBP1, pCB102, and pCD6) than in *A. wieringae* JM, and lower than in *A. wieringae*^T^.

### Validation of the vector pMTL83151 for plasmid-based expression in *Acetobacterium* strains

**Fig. 4:**
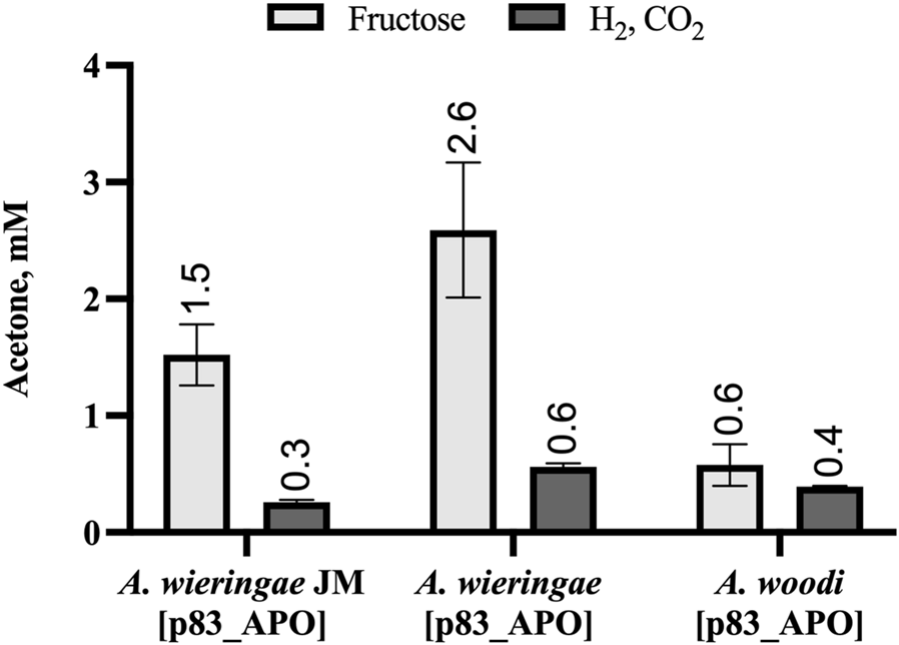
Acetone production in recombinant *Acetobacterium* derived from different host strains using fructose or H_2_/CO_2_ as substrate. *Acetobacterium* [p83_APO] strains were cultivated in serum bottles under autotrophic and heterotrophic conditions. Data represent the mean with SDs of three biological replicates

To test the heterologous production of chemicals in *Acetobacterium* strains via plasmid-based expression, as a proof of concept, we constructed the biosynthesis pathway for acetone production from acetyl-CoA in *Acetobacterium* by utilizing the shuttle vector pMTL83151. Acetone production in *Acetobacterium,* from acetyl-CoA is theoretically feasible with the presence of the acetone production operon (APO) from *Clostridium acetobutylicum* DSM 792*,* which consists of the genes *thlA* (encoding thiolase A), *ctfA/ctfB* (encoding CoA transferase), and *adc* (encoding acetoacetate decarboxylase). The APO was cloned into the pMTL83151 vector, under the control of the *thlA* native promoter, P_thlA_, and the transcriptional terminator from downstream of the CD0164 ORF of *Clostridium difficile* strain 630. The resulting plasmid, p83_APO was transformed in *A. wieringae* JM, *A. wieringae*^T^, and *A. woodii*^T^. Acetone production was detected via gas chromatography in the three recombinant *Acetobacterium* strains after 10 days of uncontrolled batch experiments under autotrophic (220 kPa 80% H_2_, 20% CO_2_) and heterotrophic (30 mM fructose) conditions (Fig. 4), confirming the expression of recombinant genes. From heterotrophic growth, *A. wieringae* JM [p83_APO] produced 1.5 ± 0.3 mM acetone, *A. wieringae* [p83_APO] 2.6 ± 0.6 mM, and *A. woodii* [p83_APO] 0.6 ± 0.2 mM, respectively. When grown on H_2_ + CO_2_, acetone production was lower (*A. wieringae* JM [p83_APO]: 0.26 ± 0.02 mM; *A. wieringae* [p83_APO]: 0.56 ± 0.03 mM; *A. woodii* [p83_APO]: 0.39 ± 0.01 mM). In the control experiments using wild-type strains and mutant strains carrying the empty vector pMTL83151, acetone could not be detected.

## Discussion

This study provides a reliable and reproducible electrotransformation protocol for use in the new acetogen *Acetobacterium wieringae* strain JM, but also applicable to the type strains of *A. wieringae* and *A. woodii*, and potentially to other acetogenic strains. We achieved robust E_T_ values from ~ 1.0 × 10^2^ up to 2.0 × 10^3^ CFU/μg_DNA_ (Fig. 2) for plasmids with the replicons pBP1, pCB102, and pCD6 in all three *Acetobacterium* strains, which are within the E_T_ range of the same plasmids in other acetogenic species (Table 3). This reinforces the transferability of Clostridial molecular tools to other acetogenic bacteria and uncovers multiple replicons for the manipulation of *A. wieringae*, which allows the diversification of applicable genetic engineering strategies. For instance, the use of two plasmids with different compatible replicons is common practice for the simultaneous replication of genes in a host, leading to increased size and number of exogenous genes expressed at once [45, 46]. The high transformation efficiencies achieved here also enable library-based approaches, which require high frequencies, in contrast to the conventional transfer of individual plasmids.

**Table 3:** Summary of electrotransformation efficiencies for different acetogens using the replicons pBP1, pCB102, pCD6 and pIM13 with the plasmid vectors pMTL82151, pMTL83151, pMTL83141, pMTL84151, and pMTL85151

Plasmid	Replicon	Acetogen	E_T_ (CFU/μg_DNA_) ^(a)^	References
pMTL82151	pBP1	*Clostridium acetobutylicum* DSM 792	1.4 × 10^2^	[28]
		*Clostridium acetobutylicum* DSM 792	4.6 × 10^2 (b)^	[53]
		*Eubacterium limosum* ATCC 8486	0.5 × 10^1^	[54]
		*Clostridium ljungdahlii* DSM 13528	3.8 × 10^3^	[31]
		*Clostridium ljungdahlii* DSM 13528	5.6 × 10^2 (b)^	[53]
		*Clostridium pasteurianum* DSM 756	4.0 × 10^2 (b)^	[53]
		*Clostridium perfringens* DSM 525	4.6 × 10^2 (b)^	[53]
		*Acetobacterium wieringae* strain JM	8.6 × 10^1^	This work
		*Acetobacterium wieringae* DSM 1911	1.5 × 10^2^	This work
		*Acetobacterium woodii* DSM 1030	5.1 × 10^2^	This work
pMTL83151	pCB102	*Clostridium ljungdahlii* DSM 13528	3.1 × 10^3^	[31]
		*Clostridium ljungdahlii* DSM 13528	3.2 × 10^2 (b)^	[53]
		*Clostridium pasteurianum* DSM 756	0.7 × 10^1^	[55]
		*Clostridium acetobutylicum* DSM 792	2.5 × 10^2^	[28]
		*Eubacterium limosum* ATCC 8486	1.8 × 10^1^	[54]
		*Acetobacterium wieringae* strain JM	1.6–5.0 × 10^2^	This work
		*Acetobacterium wieringae* DSM 1911	2.0 × 10^3^	This work
		*Acetobacterium woodii* DSM 1030	4.6 × 10^2^	This work
pMTL83141	pCB102	*Acetobacterium woodii* DSM 1030	4.0 × 10^5^	[32]
		*Acetobacterium woodii* lab-adapted strain	1.0 × 10^1^	[32]
pMTL84151	pCD6	*Clostridium acetobutylicum* DSM 792	8.5 × 10^2^	[28]
		*Eubacterium limosum* ATCC 8486	0.5 × 10^1^	[54]
		*Acetobacterium wieringae* strain JM	1.0 × 10^2^	This work
		*Acetobacterium wieringae* DSM 1911	1.6 × 10^3^	This work
		*Acetobacterium woodii* DSM 1030	4.6 × 10^2^	This work
pMTL85151	pIM13	*Clostridium acetobutylicum* DSM 792	2.9 × 10^2^	[28]
		*Eubacterium limosum* ATCC 8486	0.0 × 10^1^	[54]
		*Acetobacterium wieringae* strain JM	1.5 × 10^1^	This work
		*Acetobacterium wieringae* DSM 1911	9.0 × 10^1^	This work
		*Acetobacterium woodii* DSM 1030	1.5 × 10^1^	This work

^(a)^ Transformation efficiencies, number of colonies per microgram of plasmid DNA, ^(b)^ transformation efficiencies corrected for growth by OD_600_

Different replicons may replicate at different levels which can later influence copy number and gene expression. For example, in *A. woodii* it was shown that out of the four replicons pIP404, pBP1, pCB102, and pCD6, a negative impact on acetone production was observed with pCB102 [19]. However, also in *A. woodii*, the use of pCB102 led to higher lactate productivity than earlier constructions using pIP404 [47]. In *Clostridium tyrobutyricum*, high transformation efficiency was associated with high segregational stability for the replicons pBP1 and pCB102 but the same relation was not observed for the replicon pIM13 [48]. Generally, replicon stability can vary within species, influencing transformation efficiency, copy number, and segregational stability. Identifying the replicon stability is essential in guiding the replicon selection for downstream applications. While high replicon stability is essential for plasmid-based expression of recombinant proteins, replicon instability leads to easier plasmid loss from growth without selective pressure, which is central to allele-coupled exchange [49] and CRISPR–Cas9-mediated genome editing [50]. Future studies will assess the replicon stability in *A. wieringae*. As higher transformation efficiencies in *A. wieringae* JM and *A. wieringae*^T^ were obtained with the replicon pCB102, we selected the plasmid pMTL83151 for demonstration of heterologous production of acetone in *A. wieringae* JM, *A. wieringae*^T^, and *A. woodii*^T^ via plasmid-based expression of the acetone biosynthetic pathway from *C. acetobutylicum*. Acetone production was detected for all three recombinant *Acetobacterium* strains.

The investigation of various electrotransformation parameters enabled us to understand the impact of each parameter which gives flexibility in adapting the protocol to diverse plasmids and conditions, securing the success of future transformations with the different plasmids. Two factors that greatly influence the success of transformation experiments are the plating procedure and the colony-picking method. For plating, we used 50 mL of media for 1.5 mL of inoculum in 200 mL anaerobic serum bottles, using a pour-plating method described in the Materials and methods section. Most reported protocols use Petri dishes for the plating procedure, either by mixing the inoculum with molten agar [31, 53] or by spreading the inoculum on the agar [21, 29], with further incubation inside the anaerobic chamber (Protocols 2, 4–6, Table 1). In our hands, these approaches led to lower transformation efficiencies and low reproducibility when using anaerobic jars (Oxoid™ AnaeroJar™) for the incubation of Petri dishes, as we could not incubate the cells inside the anaerobic chamber. Other studies report the use of liquid media for the selection of transformants [19, 39], but even though this method led to successful transformation experiments in our hands, it required additional subculturing steps and a final plating step to obtain a genetically homogeneous population of transformants.

Even though the electrotransformation protocol was optimized for *A. wieringae* JM, lower transformation efficiencies were obtained for this strain compared to the type strains *A. wieringae* and *A. woodii*. This may be explained by single-nucleotide polymorphisms (SNPs) which may have occurred during the enrichment and isolation of *A. wieringae* JM [25]. For instance, SNPs were identified in a lab-adapted strain of *A. woodii* which were associated with lower transformation efficiencies when compared to the type strain *A. woodii* DSM 1030 [32]. In addition, different restriction-modification (RM) systems in *Acetobacterium* strains may exhibit different protection against foreign DNA. All three *Acetobacterium* strains have RM systems that target and cleave unmethylated adenosine such as the HsdS-HsdM-HsdR system [51]: *A. woodii*^T^ (H6LBT5, H6LHR7, H6LHR4, UniProtKB); *A. wieringae*^T^ (A0A1F2PIJ0, A0A1F2PL45, A0A1F2PJC8, UniProtKB); *A. wieringae* JM (A0A5D0WQE5, A0A5D0WKG3, UniProtKB). However, only in *A. woodii*^T^ and *A. wieringae*^T^ RM systems targeting and cleaving unmethylated cytosine could be found, such as the Dcm, ydiP, and aplIM systems (H6LJP1, H6LIV2, A0A1F2PM82, A0A1F2PKZ9, UniProtKB). Additionally, a type II restriction endonuclease from the NgoFVII family, which recognizes the double-stranded sequence ‘GCSGC’ and cleaves after G-4 could be found in *A. wieringae* JM (A0A5D0WXZ3, UniProtKB), but not in *A. woodii*^T^ and *A. wieringae*^T^. With the use of the *E. coli* TOP10 strain for plasmid preparation, which provides Dam and Dcm methylation, protection against the RM systems described above should be secured in the type strains of *A. woodii* and *A. wieringae*. Since *A. wieringae* JM does not methylate DNA on the cytosine, the use of an *E. coli* strain without Dcm for plasmid preparation may improve transformation efficiencies in *A. wieringae* JM, as observed in *C. ljungdahlii* [31] and *C. autoethanogenum* [52].

## Conclusions

In this work, the optimization of competent cell preparation, electroporation parameters, and plating procedure enabled the electrotransformation of *A. wieringae* strain JM, *A. wieringae* DSM 1911^ T^, and *A. woodii* DSM 1030^ T^ at efficiencies of up to 2.0 × 10^3^ CFU/μg_DNA_. The work here also demonstrates the establishment of a molecular toolkit in *A. wieringae*, currently comprising the thiamphenicol selection marker *catP* and four Gram + origins of replication (pBP1 from *C. botulinum*, pCB102 from *C. butyricum*, pCD6 from *C. difficile*, and pIM13 from *B. subtilis*). This is the first report of a genetic transformation procedure for *A. wieringae* and represents a key advancement for this industrially C1-gas fermenting relevant species with important applications for renewable chemical and biofuel production.

## Materials and methods

### Bacterial strains and growth conditions

Bacterial strains and plasmids used in this study are listed in Table 4. *E. coli* was routinely grown at 37 °C or 30 °C in LB broth or on LB agar. *E. coli* TOP10 was used for plasmid propagation and *E. coli* CA434 as a conjugative donor strain. *C. acetobutylicum* was grown at 37 °C under strictly anaerobic conditions using the DSMZ medium 104b. *Acetobacterium* strains were grown anaerobically at 30 °C in basal medium prepared as described previously [56], with the addition of yeast extract (0.5 g/L), d-fructose (5 g/L), and phosphate buffer, pH 7.0 (K_2_HPO_4_/KH_2_PO_4_, 10 mM). All basal media were extensively flushed with N_2_ and residual oxygen was removed with ~ 0.8 mmol L^−1^ sodium sulfide (Na_2_S.7-9H_2_O) as a reducing agent [56]. Media were supplemented with antibiotics, when necessary, in the following concentrations: for *E. coli*, chloramphenicol (25 μg/mL) and kanamycin (50 μg/mL); for *Acetobacterium* strains, thiamphenicol (15 μg/mL).

**Table 4:** Bacterial strains and plasmids used in this work

Strain or plasmid	Relevant characteristics	Source
Strains		
* Acetobacterium wieringae* strain JM	Wild type	Lab stock
* Acetobacterium wieringae* DSM 1911^ T^	Wild type	DSMZ collection
* Acetobacterium woodii* DSM 1030^ T^	Wild type	DSMZ collection
* Clostridium acetobutylicum* DSM 792^ T^	Wild type	DSMZ collection
* Escherichia coli* DH5α	*fhu*A2∆(*arg*F-*lac*Z)U169 *pho*A *gln*V44 Φ80 ∆(*lac*Z)M15 *gyr*A96 *rec*A1 *rel*A1 *end*A1 *thi*-1 *hsd*R17	NZYTech
* Escherichia coli* TOP10	F- *mcrA* Δ(*mrr-hsd*RMS-*mcr*BC) Φ80*lac*ZΔM15 Δ*lac*X74 *rec*A1 *ara*D139 Δ(*ara-leu*)7697 *gal*U *gal*K *rps*L (StrR) *end*A1 *nup*G	Invitrogen
* Escherichia coli* CA434	*E. coli* HB101 containing plasmid R702 thi-1 hsdS20 (r-B, m-B) supE44 recAB ara-14 leuB5proA2 lacY1galK rpsL20 (strR) xyl-5 mtl-1	[28]
Plasmids		
R702	Tra^+^, Mob^+^ conjugative plasmid	[28]
pMTL82151	Gram^+^: pBP1, *catP*; Gram^−^: ColE1, *catP*; application: MCS	[28]
pMTL83151	Gram^+^: pCB102; *catP*; Gram^−^: ColE1, *catP*; application: MCS	[28]
pMTL84151	Gram^+^: pCD6; *catP*; Gram^−^: ColE1, *catP*; application: MCS	[28]
pMTL85151	Gram^+^: pIM13, *catP*; Gram^−^: ColE1, *catP*; application: MCS	[28]
pMTL83_APO	pMTL83151, P_thlA_, *thlA*, *ctfAB*, *adc* from *C. acetobutylicum*	This study

For the growth of *Acetobacterium* strains on solid media, a pour-plating method was used. In detail, basal media supplemented with 1.5 g/L yeast extract, was prepared with 1.3% agar and aliquoted by 45 mL in 200 mL serum bottles (Glasgerätebau Ochs, Bovenden, Germany) for anaerobic cultivation. Bottles were then flushed with 100% N_2_, pressurized to 100 kPa, and sterilized. After this, media were cooled down to 40–50 °C and then mixed with vitamins, d-fructose (5 g/L), reducing agent, antibiotics, and 1.5 mL of liquid bacterial culture. After solidification, bottles were filled up with 20% CO_2_ and 80% N_2_ to a final pressure of 170 kPa. The bottles were then incubated at 30 °C until single colonies were visible (3–10 days). Colonies were picked inside the anaerobic working station with long wooden toothpicks.

Recombinant *E. coli* stocks were stored at − 80 °C in 25% glycerol, and *Acetobacterium* wild type and recombinant stocks were prepared by concentrating 10 × cultures in fresh basal media (OD_600_ of 0.30–0.80) and stored at − 80 °C with 10% DMSO.

In the case of batch experiments to detect acetone production in recombinant *Acetobacterium* strains, cells were cultivated in 200 mL serum bottles with 50 mL of basal media, as described above, using thiamphenicol with a final concentration of 25 μg/mL. For heterotrophic growth fructose was added at a final concentration of 30 mM under the headspace of 80% N_2_ and 20% CO_2_ (*v*/*v*) at 220 kPa. For autotrophic growth, the headspace was set at 220 kPa with 80% H_2_ and 20% CO_2_ (*v*/*v*). Recombinant strains were previously adapted autotrophy by subculturing each clone three times in fresh medium before the growth experiment.

### Conjugative plasmid transfer

Conjugative transformation of *A. wieringae* strain JM was performed as described previously [28], with a few modifications. Donor cells of *E. coli* CA434 carrying the plasmids pMTL82151, pMTL83151, pMTL84151, and pMTL85151, previously transformed by heat-shock transformation, were collected by centrifugation of 1 mL of antibiotic-supplemented overnight culture at 4000 × g for 2 min. Donor cells were washed once with PBS, then the pellet was gently resuspended in 1 mL of 2 times concentrated overnight culture of the *A. wieringae* JM recipient. This mating mixture was spotted onto a non-selective plate of basal media and incubated anaerobically for 24 h at 30 °C without inverting the plates. The mating mixture was resuspended from the surface of the plate in 2 mL of PBS using a spreader and transferred into 50 mL of selective (25 μg/mL thiamphenicol) liquid basal medium without fructose nor yeast extract, and grown autotrophically on syngas (60% CO, 30% H_2_, 10% CO_2_; (*v*/*v*)) till growth was obtained. Cultures were then sub-cultured four times under the same conditions. To identify the presence of *E. coli*, 0.2 mL of the latest culture was spread on LB agar and grown aerobically.

### DNA manipulation

E. coli DH5α (NZYTech, Lisbon, Portugal) cells were used for plasmid construction and storage. *E. coli* TOP10 cells (Invitrogen, MA, USA) were used for plasmid preparation before electrotransformation in *Acetobacterium*. Plasmids were then isolated from *E. coli* using the GenElute^™^ plasmid miniprep kit (Sigma-Aldrich, MO, USA). For *Acetobacterium* transformants, plasmid DNA was extracted and purified using also the GenElute^™^ plasmid miniprep kit (Sigma-Aldrich, MO, USA) after the following pre-treatment: 10 mL of late-exponential phase cells were collected by centrifugation (4000 × g, 15 min, 4 °C), resuspended in 1 mL of 10 mM Tris–NaCl, pH 8, with the addition of 10 mg of lysozyme and incubated at 37 °C for 1 h. For genomic DNA extraction from *C. acetobutylicum*, twenty milliliters of culture were collected by centrifugation (4000 × g, 15 min, 4 °C) and used in the FastDNA SPIN kit for soil (MP Biomedicals, Solon, OH, USA). Restriction enzymes for DNA digestion, T4 DNA ligase for DNA ligation, and Phusion High-Fidelity DNA Polymerase for amplification of DNA fragments were purchased from New England Biolabs GmbH (Main, Germany), Promega Corporation (WI, USA), and Thermo Fisher Scientific (Waltham, MA, USA), respectively. The QIAquick PCR Purification Kit (Qiagen, Valencia, CA, USA) was used to purify the PCR products and DNA fragments obtained by treatment with restriction enzymes. Gel extraction was performed using the QIAquick Gel Extraction Kit (Qiagen, Valencia, CA, USA). All primers were synthesized by Eurofins Genomics (Ebersberg, Germany).

All primers used to construct plasmids for acetone production are listed in Table 5. The acetoacetate decarboxylase gene (*adc*; CA_P0165), the acetoacetyl-CoA:acetate/butyrate CoA transferase genes (*ctfA/ctfB*; CA_P0163/CA_P0164), and the thiolase gene (*thlA*; CA_C2873) with its promoter region (P_thlA_) were obtained from *C. acetobutylicum* via PCR amplification using each respective primer pair. Amplified genes were sequentially cloned into the MCS of vector pMTL83151 using the restriction enzymes NotI-HF, KpnI-HF, BamHI-HF, and SalI-HF to form the plasmid p83_APO.

**Table 5:** Primers used in this study

Primer^a^	Sequence (5´- 3´)^a^	Description
SalI_PthlA_fw	GCCGACGCGTCGACATATTGATAAAAATAATAATAGTGGG	Amplification of P_thlA__*thlA*
BamHI_thlA_rev	CGGGATCCCTAGCACTTTTCTAGCAATATTGC	
BamHI_ctfA_fw	CGACCGGGATCCATGAACTCTAAAATAATTAG	Amplification of *ctfAB*
KpnI_ctfB_rev	GGGGTACCCTAAACAGCCATGGGTCTAAG	
KpnI_adc_fw	GAGGGGTACCATGTTAAAGGATGAAGTAATTAAAC	Amplification of *adc*
NotI_adc_rev	GGCCGCGGCCGCTTACTTAAGATAATCATATATAAC	
pCB102_mid_rev	CTGTTATGCCTTTTGACTATCAC	Plasmid verification of constructs
M13_rev	CAGGAAACAGCTATGAC	

^(a)^ Restriction sites are bold

### Preparation of electrocompetent cells and electrotransformation

For the preparation of electrocompetent cells of *Acetobacterium* strains using the optimized protocol, 0.5 L of basal media supplemented with 40 mM d-threonine were inoculated with − 80 °C culture stocks to an OD_600_ of 0.05 to 0.1 and incubated at 30 °C overnight. At an OD_600_ range of 0.3–0.6, cultures were placed on ice for 20 min and brought into an anaerobic workstation (Coylab, MI, USA) under a gas atmosphere of about 5% H_2_ and 95% N_2_ (*v*/*v*). The bottles of the cultures were then opened and stepwise harvested in pre-deoxygenated 50 mL tubes by centrifugation (4000 × g, 15 min, 4 °C), and cell pellets were washed once or twice with 50 mL ice-cold SMP buffer pH 6 (270 mM sucrose, 1 mM MgCl_2_, 7 mM NaH_2_PO_4_). Subsequently, the washed cells were resuspended in SMP buffer 10% DMF pH 6, to a final OD_600_ of 30, and aliquot by 2 mL in anaerobic vials and stored at − 80 °C.

For electroporation, competent cells were thawed on ice and brought into an anaerobic workstation. Electroporation cuvettes with a gap width of 0.2 cm (Bio-Rad, CA, USA) were placed on ice, and 1–3 μg of plasmid DNA were added, following 0.2 mL of competent cells. Electroporation was performed at the following conditions: 2.0 kV, 20 Ω, and 25 μF (*E. coli* Pulser^™^ Transformation Apparatus, Bio-Rad, CA, USA). Directly after electroporation, cells were transferred into 3 mL of basal media and incubated for 20 h at 30 °C. After this non-selective outgrowth step, 1.5 mL of culture were plated using the pour-plating method described above. The transformants were further confirmed by plasmid digestion analysis or PCR amplification. For colony picking, long wooden toothpicks produced the best results, while the use of the pointy end of plastic inoculation loops, long pipette tips, or metallic long needles resulted in lower ratios of grown/picked colonies. The porous surface of wooden toothpicks and their thin pointy ends allow better accuracy and a sticking effect when picking colonies submerged in agar plates. During plating, appropriate dilutions should be done to obtain plates in fewer colonies (Additional file 1: Fig. S1c), and therefore facilitate colony picking, reducing contamination risks and promoting the homogeneity of clones.

### Acetone measurement

The concentration of acetone was analyzed by use of a gas chromatograph (Varian 4000, Agilent Technologies, CA, USA) equipped with a flame ionization detector (FID), an autosampler (CP-8400, Bruker, MA, USA), and a Teknokroma Meta.WAX 30 m × 0.25 mm × 0.25 mm TR-810232 capillary column. For sample preparation for GC analysis, 1-butanol was added as an internal standard to cell-free supernatant, in a working concentration of 5 mM. For detection, 10 μL of the sample were injected at a split ratio of 10. The injector temperature was 120 °C, and the column oven temperature was held at 35 °C for 5 min and then increased to 120 °C at a rate of 15 °C/min. The detector temperature was set at 160 °C. The carrier gas was N_2_ at a flow rate of 1 mL/min.

## Supplementary Information

**Additional file 1: Figure S1.** Agar plates in anaerobic serum bottles for selection of *A. wieringae *JM transformants. (a) Overview of serum bottles after inoculation. Plates are inoculated outside of the anaerobic chamber using syringes while agar temperature is around 40 -50 °C. After solidification of the agar, and before incubation at 30 °C, bottles are pressurized with 170 kPa of 80 % N_2_ and 20 % CO_2_ (v/v). (b) Visualization of colonies after 5 days of incubation using 1.5 mL of *A. wieringae *JM electroporated cells with the plasmid pMTL83151. (c) Visualization of colonies after 5 days of incubation using 0.2 mL of *A. wieringae *JM electroporated cells with the plasmid pMTL83151.

## Data Availability

All data generated or analyzed during this study are included in this published article and/or its supplementary information files. The data that support the findings of this study are available from the corresponding author upon reasonable request.

## Abbreviations

EU: European Union
GHG: Greenhouse-gas
*A. wieringae* JM: *Acetobacterium wieringae* Strain JM
MIC: Minimum inhibitory concentration
SMP: Sucrose-magnesium-phosphate
DSMO: Dimethyl sulfoxide
DMF: Dimethylformamide
Dcm: Cytosine methyltransferase
Dam: Adenine methyltransferase
E_T_: Transformation efficiency
CFU: Colony-forming units
RM: Restriction modification
OD_600_: Optical density at 600 nm
MCS: Multi-cloning site
DSMZ: German Collection of Microorganisms and Cell Cultures
APO: Acetone production operon
SNPs: Single-nucleotide polymorphisms
