# Supplementary material for: Developing a genetic engineering method for Acetobacterium wieringae to expand one-carbon valorization pathways
Source: Biotechnol Biofuels Bioprod. 2023 Feb 14;16:24. doi: 10.1186/s13068-023-02259-6 (PMC9930230; doi:10.1186/s13068-023-02259-6)
Supplement: Supplementary file 1 — Additional file 1: Figure S1. Agar plates in anaerobic serum bottles for selection of A. wieringae JM transformants. (a) Overview of serum bottles after inoculation. Plates are inoculated outside of the anaerobic chamber using syringes while agar temperature is around 40 -50 °C. After solidification of the agar, and before incubation at 30 °C, bottles are pressurized with 170 kPa of 80 % N2 and 20 % CO2 (v/v). (b) Visualization of colonies after 5 days of incubation using 1.5 mL of A. wieringae JM electroporated cells with the plasmid pMTL83151. (c) Visualization of colonies after 5 days of incubation using 0.2 mL of A. wieringae JM electroporated cells with the plasmid pMTL83151. [file 13068_2023_2259_MOESM1_ESM.docx]

**Additional file 1**

Developing a genetic engineering method for *Acetobacterium wieringae* to expand one-carbon valorization pathways

João P. C. Moreira^1,2^, John T. Heap^3^, Joana I. Alves^1,2^, Lucília Domingues^1,2*^

^1^CEB – Centre of Biological Engineering, University of Minho, 4710-057 Braga, Portugal ^2^LABBELS – Associate Laboratory, Braga/Guimarães, Portugal

^3^School of Life Sciences, University of Nottingham, Biodiscovery Institute, University Park, Nottingham, NG7 2RD, UK

*Corresponding author: Lucília Domingues

Tel: +351253604405

Email: luciliad@deb.uminho.pt

**Running title:** Developing a genetic engineering method for *Acetobacterium wieringae*


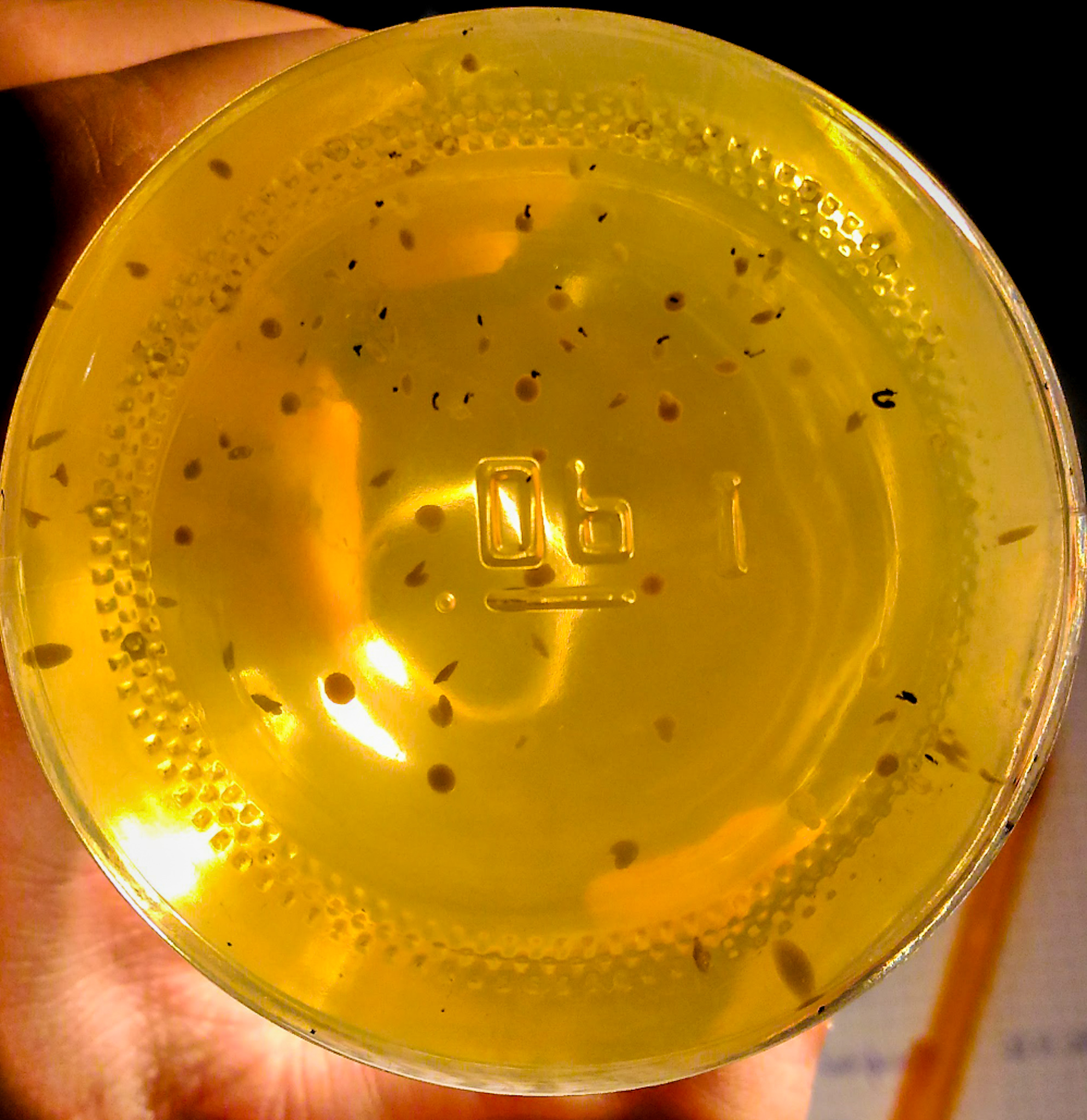

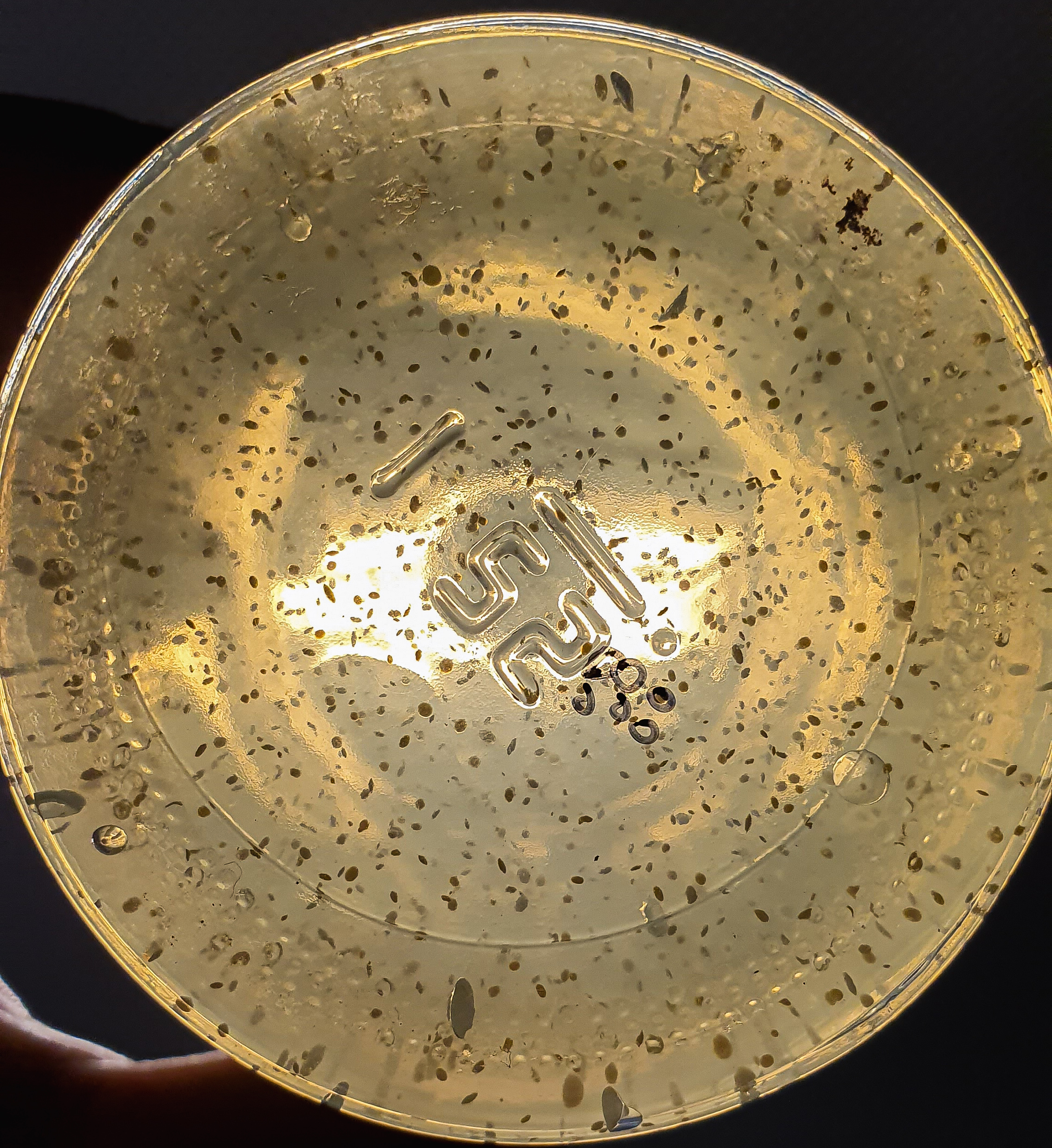


**(a)**

**(b)**

**(c)**


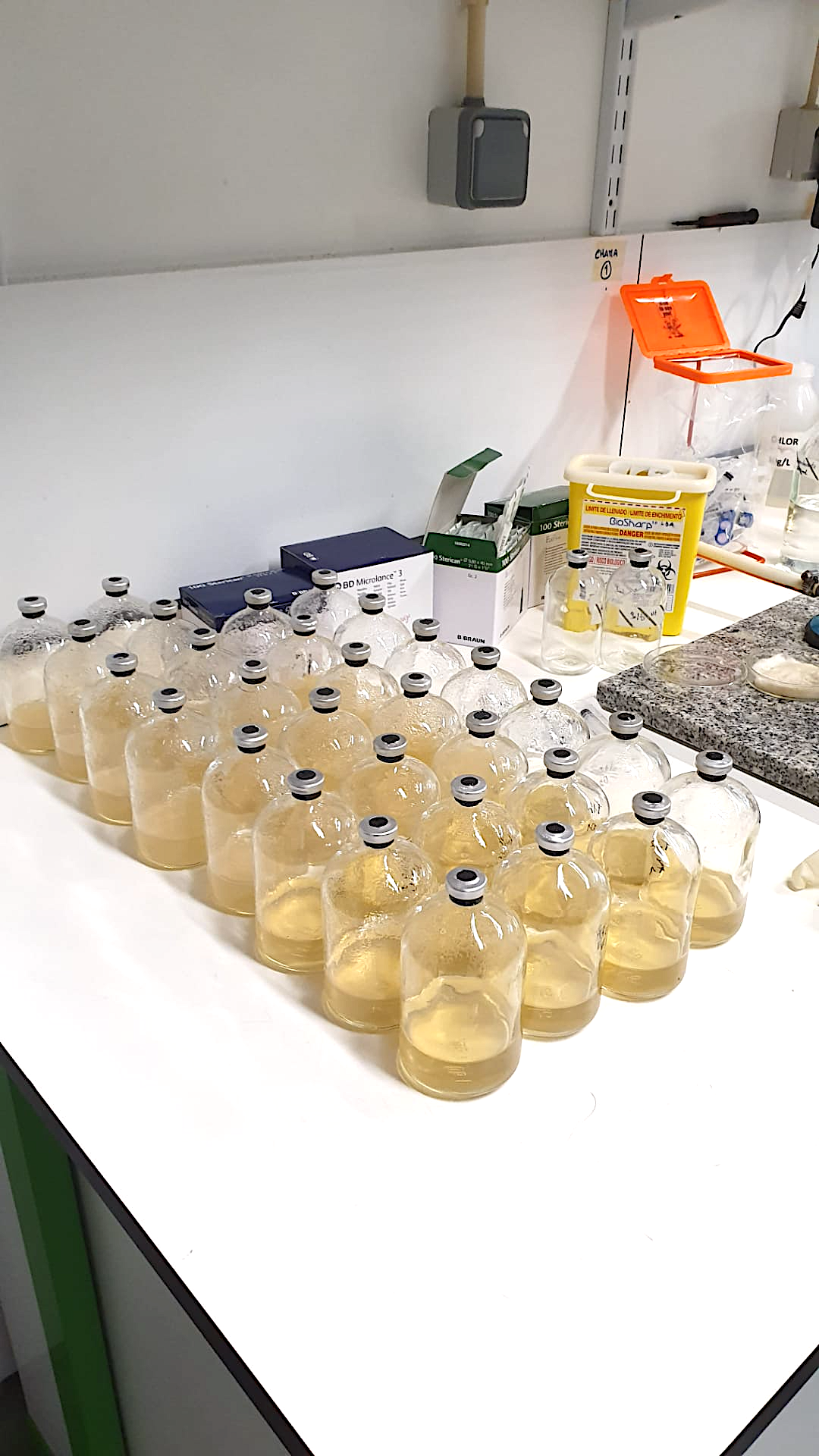


**Fig. S1** – **Agar plates in anaerobic serum bottles for selection of *A. wieringae* JM transformants. (a)** Overview of serum bottles after inoculation. Plates are inoculated outside of the anaerobic chamber using syringes while agar temperature is around 40 -50 °C. After solidification of the agar, and before incubation at 30 °C, bottles are pressurized with 170 kPa of 80 % N_2_ and 20 % CO_2_ (v/v). **(b)** Visualization of colonies after 5 days of incubation using 1.5 mL of *A. wieringae* JM electroporated cells with the plasmid pMTL83151. **(c)** Visualization of colonies after 5 days of incubation using 0.2 mL of *A. wieringae* JM electroporated cells with the plasmid pMTL83151.
